# The Secret Family Life of a Group of Golden Jackals on Samos, Greece

**DOI:** 10.1002/ece3.70620

**Published:** 2024-12-06

**Authors:** Jonas Custers, Jennifer Hatlauf, Sem van der Niet, Beatriz Tintoré, Anastasia Miliou

**Affiliations:** ^1^ Archipelagos Institute of Marine Conservation Research Base Samos Greece; ^2^ Department of Animal Behaviour and Cognition Utrecht University Utrecht The Netherlands; ^3^ Institute of Wildlife Biology and Game Management, Department of Integrative Biology and Biodiversity Research (DIBB) BOKU University Vienna Vienna Austria

**Keywords:** behavioural ecology, camera trapping, Canis aureus, individual identification, social group relationships, social network analysis, social system

## Abstract

The golden jackal ( *Canis aureus* ) is remarkably flexible in terms of behaviour. This is advantageous to the range expansion of the species to northern and western Europe. Despite the widespread distribution of the golden jackal, many aspects of its behaviour are still poorly known. In this study, we have aimed to improve our general understanding of golden jackal social behaviour by monitoring one family group of a unique insular population living on Samos (Greece) using camera trap data over a study period of 9 months. Successful identification of individual golden jackals based on visual characteristics, determination of the dominance hierarchy and social network analyses has allowed us to gain insights into the group's social organisation, mating system and social structure determined by social relationships. We revealed the studied family group to be relatively stable, consisting of a dominant adult pair and one or two generations of their offspring. Some major changes occurred during the breeding season in terms of social behaviour, group composition and structure. A total of six pups were born, which were cared for by both dominant adults as well as one male and one female yearling who stayed as helpers at the nest. Both the dominant female and the female yearling showed signs of lactation, suggesting either a case of pseudopregnancy or allonursing. Using non‐invasive methods combined with individual identification based on coat colouration patterns, this research contributes to our understanding of the social behaviour of the golden jackal population on Samos in Europe and, by extension, of the species as a whole.

## Graphical abstract

This study provides insights into the social behaviour of golden jackals on Samos Island (Greece), highlighting the group's composition, structure and dynamics. By monitoring one group over 9 months with camera traps, we identified a stable family unit led by a dominant pair, with their offspring and helpers playing key roles. The findings reveal changes in social behaviour during the mating season, contribute insights into the species' overall social system and emphasise the importance of non‐invasive methods for studying wildlife populations.

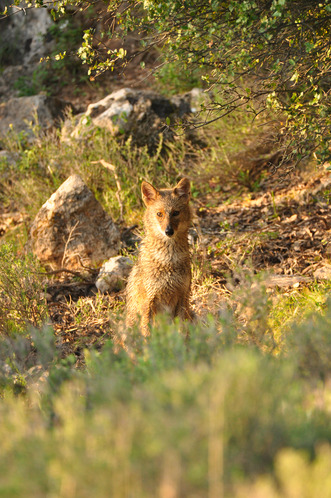

## 1 Introduction

**FIGURE 1:**
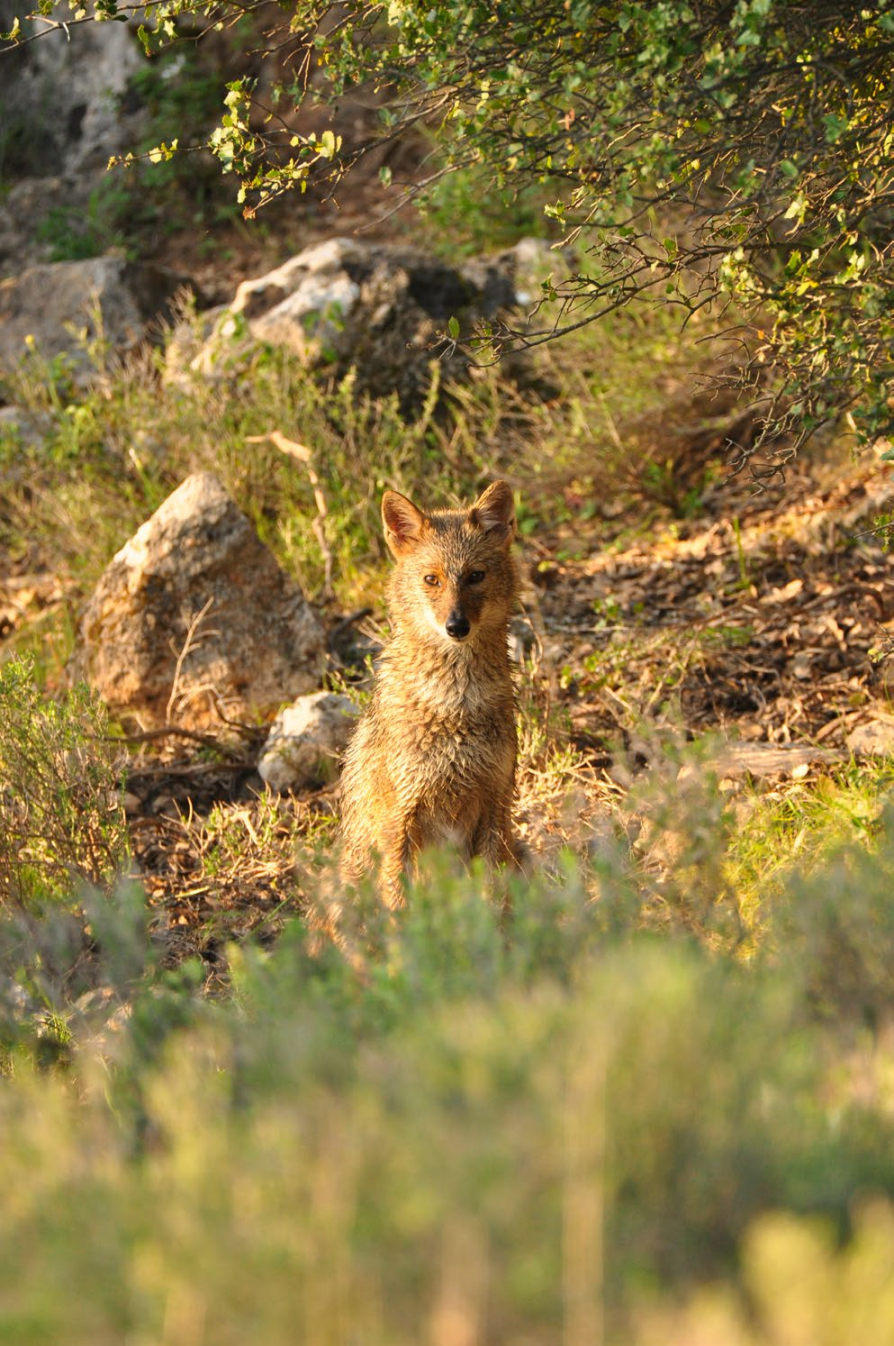
Golden jackal cover image. The individual in the picture is ‘EC’, the male helper.

Golden jackals ( *Canis aureus* ; Figure 1) have been surprising researchers over the past decade with a range expansion originating from the Balkans north‐ and westwards throughout Europe (Hatlauf, Bayer et al. 2021; Stefanović et al. 2024; Trouwborst, Krofel, and Linnell 2015). Currently, established populations can be found throughout Southeast and Central Europe (Hatlauf, Bayer et al. 2021). However, records have occurred as far west as Spain and France (Bouchet 2017; Buruaga et al. 2023; Royo‐Vicente and García 2024) and as far north as Finland and the Subarctic and Arctic regions in Norway and Russia (Sørensen and Lindsø 2021, Rykov, Kuznetsova, and Tirronen 2022). This natural range expansion has raised many questions on why golden jackals have suddenly become such a successful species in Europe (Hatlauf, Bayer et al. 2021; Hatlauf, Böcker et al. 2021; Krofel et al. 2017).

It is commonly agreed upon that this range expansion illustrates the species' behavioural flexibility (Ćirović, Penezić, and Krofel 2016; Hatlauf, Bayer et al. 2021; Kebede 2017; Kojola et al. 2024; Moehlman and Hayssen 2018). Yet, current knowledge of the behavioural ecology of wild golden jackals in Europe is limited, mostly due to the elusive nature of the species (Csányi et al. 2023; Pecorella et al. 2023). However, this knowledge is crucial for developing (cost‐)effective monitoring methods, identifying potential future human–animal conflicts and supporting coexistence (Berger‐Tal and Saltz 2016; Bro‐Jørgensen, Franks, and Meise 2019). One population of golden jackals holding great research potential is the insular population inhabiting Samos, a Greek island located in the eastern Aegean and separated by only a narrow sea strait (±2 km) from the Anatolian mainland (Giannatos 2004; Giannatos et al. 2005). Due to their relative isolation from the mainland, the population on Samos has likely been subjected to several short‐term diversification processes caused by reduced rates of colonisation and gene flow (Blakeslee et al. 2019; Matthews and Triantis 2021). Like in mainland Greece, golden jackals on Samos faced a rapid population decline during the end of the last century. However, legal protection has resulted in a likewise rapid recovery of most populations both on Samos and on the mainland concurring with the global population range expansion of the species (Giannatos 2004; Giannatos et al. 2005; Karamanlidis et al. 2023).

The origins of the golden jackal population on Samos are not well known, since the native range of the species is yet to be determined (Stefanović et al. 2024). Nevertheless, a study on the genetic structure has found that there have been at least some connections between Samos and mainland Greece and that the genetic structure of the population on Samos is highly differentiated from populations on the mainland (Rutkowski et al. 2015). This indicates that the geographic barrier formed by the sea has resulted in a genetic distinctiveness of golden jackals living on Samos from those living on the mainland. Golden jackals on Samos Island are relatively easy to monitor due to their relatively higher population density than that in the mainland (Giannatos et al. 2005). Nonetheless, only a handful of past research efforts have been made regarding the behavioural ecology of golden jackals on Samos (Bulmer 2015; Pietroluongo, Leggett et al. 2018; Pietroluongo, Linardaki et al. 2018).

Acquiring information on golden jackal social behaviour is challenging, as this requires distinction between individuals. However, with close observations, individual golden jackals can be distinguished based on coat colouration patterns (Macdonald 1979; Wandrey 1975). Generally, golden jackals live in close family groups consisting of a monogamous dominant pair and their offspring (Macdonald 1979). By engaging in intimate social interactions, group members reinforce their social bonds and establish the social hierarchy. They cooperatively secure food sources and fend off strangers from their group's territory. Yearlings are known to sometimes remain at the nest as helpers, meaning that they aid their parents in raising subsequent generations of offspring before dispersing (Lanszki et al. 2018; Macdonald 1979; Wandrey 1975). Only recently, in one social unit, allosuckling behaviour, or the suckling of pups by a female other than the biological mother, has been documented, which may have been due to the breeding of two females within one social group (Pecorella et al. 2023).

**FIGURE 2:**
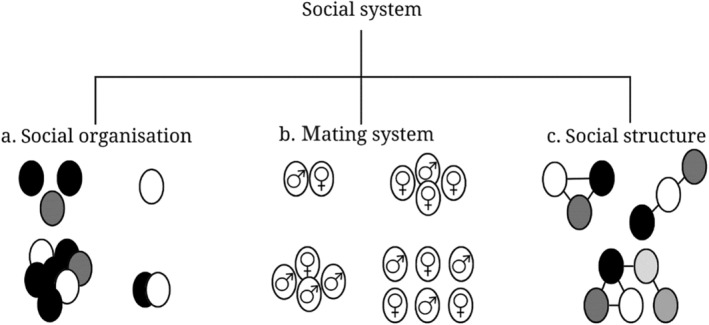
The three components of a social system with (a) social organisation: size, composition, cohesion and genetic structure of a social unit; (b) mating system: who mates with whom and how often; (c) social structure: the sum of all social relationships (after Kappeler et al. 2013; Custers 2022).

We studied the social system of the golden jackals on Samos. A social system consists of three interrelated components: social organisation, mating system and social structure (Kappeler et al. 2013) (Figure 2). First, the social organisation of a group is determined by aspects such as its size, (genetic) composition and cohesion (Kappeler et al. 2013). Second, the mating system depends on the number of breeding males and females within a social group, and how often these individuals mate (Clutton‐Brock 1989). Third, a group's social structure is the emergent outcome of all social interactions (except mating) and relationships within the group. Since social interactions and relationships strongly vary between individuals, and even within individuals throughout their lives, the social structure of a group may vary over a longer time (Hinde 1976; Kappeler et al. 2013). This knowledge is pivotal for understanding the ongoing range expansion of the species, as well as for developing effective monitoring and conservation methods.

In this study, we aimed to gain insights into the social behaviour of one group of golden jackals by exploring each of these three components. By closely monitoring this group, we planned to identify the group members and subsequently look at their social interactions and the strength of the social bonds between them. We hypothesised that individual golden jackals can be distinguished based on their unique coat colourations (Macdonald 1979). We also hypothesised that the studied group consisted of an adult pair and their offspring (Macdonald 1979) and, furthermore, expected changes in composition over time, as well as variations in behaviour depending on the individual's position within the family (Macdonald 1979; Moehlman 1987; Wandrey 1975). We explored the intricate social lives of these golden jackals by means of individual recognition based on camera trap pictures, the subsequent determination of the dominance hierarchy and social network analyses. Presented work contributes to narrowing the research gap on golden jackal social behaviour.

## 2 Materials and Methods

### 2.1 Study Area

**FIGURE 3:**
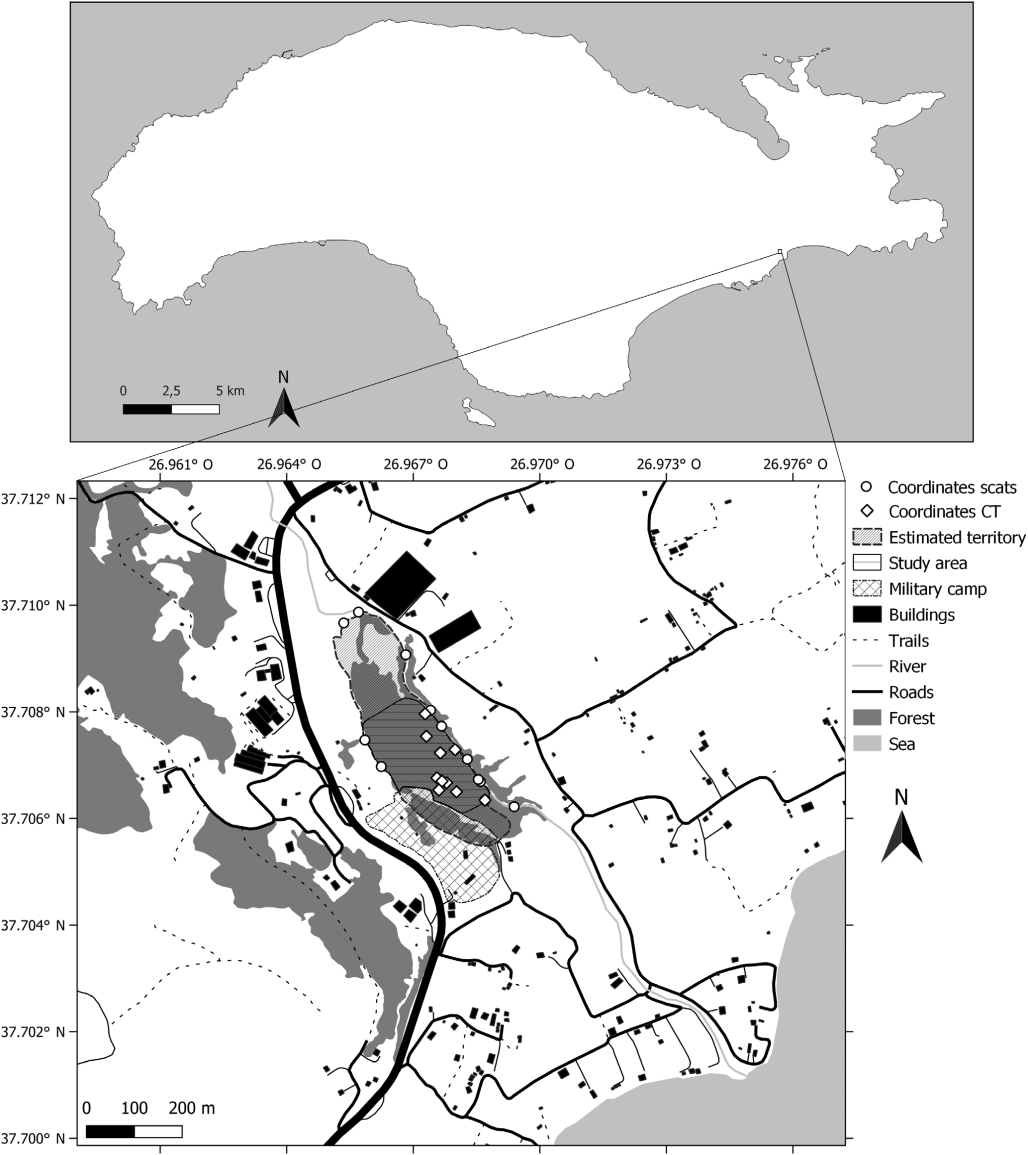
Map of the study area in Potami Mesokampos in the southeast of Samos Island, Greece. Territory borders have been roughly estimated based on the locations of scats found furthest away from the suspected core.

This study was conducted on the Greek island of Samos over a period of 9 months. Located in the eastern Aegean Sea and less than 1.7 km from the Anatolian mainland, Samos has a length of about 45 km and covers a total surface area of approximately 480 km^2^ (Stiros et al. 2000). The study area is located in Potami Mesokampos (Ποτάμι Μεσοκάμπου) in the southeast of Samos and has a total size of approximately 0.032 km^2^. This area was selected based on the approximate estimation of the origins of group howls and the presence of golden jackal tracks. The area consists of a dried‐out riverbed carved from north to south. In the west of the riverbed are small pastures and agricultural fields surrounded by dense vegetation and patches of coniferous forest that cover the eastern hill flank. Even further to the west, the research area is bordered by a military camp. However, the old barbed‐wire fences do not form a barrier to the golden jackals. In the east of the riverbed, the landscape is dominated by agricultural fields with small farms. Parallel to the dried‐out riverbed, a small road connects a larger road in the north with houses near the coast in the south (Figure 3).

Human activity in the study area is fairly low, with only the occasional goat shepherd passing through with his herd and dogs. Similarly, activity in the military camp in the west is relatively low. Nevertheless, litter coming from households, agriculture and river deposits can be found throughout the whole research area. This includes plastics, fabrics, metals, glass, barbed wires, agricultural tools, etc. In addition, the area to the east of the study area is subjected to high‐to‐medium human activity throughout the day. Besides humans, the study area is frequently visited by domestic animals, including domestic dogs and cats. On Samos, golden jackals do not face a big risk of predation, except for large birds of prey, which may potentially catch younger individuals. Other wild mammals that do occur are wild boar ( *Sus scrofa* ), European hare ( *Lepus europaeus* ), stone martens ( *Martes foina* ), least weasel ( *Mustela nivalis* ) and small rodents.

### 2.2 Data Collection

For data collection, we used a total of four camera traps: one Camouflage EZ‐Solar Wildcamera (Camera 1: total deployment time of 322 days) and three Bushnell Trophy camera traps (total deployment time Camera 2: 119 days; Camera 3: 287 days; Camera 4: 105 days) (Table 1). Deployment times differed between camera traps due to logistical and technical issues. Two different types of cameras were used. The first camera was equipped with low‐glow infrared lighting, meaning that an infrared light is emitted at night that is visible to wildlife. This results in high‐resolution recordings at night that are useful for individual identification. Meanwhile, the latter three camera traps were equipped with invisible no‐glow infrared lights, sacrificing resolution for capturing nighttime recordings without the risk of alerting the animals.

**TABLE 1:** Details on the locations and periods of deployment of the camera traps used in this study. No data have been collected in May due to logistical issues.

Device	Coordinates	Deployment period	Observation time
Camouflage EZ‐Solar Wildcamera	37.7080 N, 26.9673 E	12‐12‐2023 to 30‐12‐2023	325 s
	37.7065 N, 26.9680 E	30‐12‐2023 to 21‐02‐2023	1028 s
	37.7067 N, 26.9677 E	21‐02‐2023 to 18‐04‐2023	1618 s
	37.7072 N, 26.9677 E	14‐09‐2023 to 30‐10‐2023	2490 s
Bushnell Trophy camera 1	37.7067 N, 26.9678 E	16‐01‐2023 to 18‐04‐2023	1816 s
	37.7073 N, 26.9680 E	27‐09‐2023 to 24‐10‐2023	339 s
Bushnell Trophy camera 2	37.7064 N, 26.9687 E	16‐01‐2023 to 30‐10‐2023	4303 s
Bushnell Trophy camera 3	37.7074 N, 26.9678 E	16‐01‐2023 to 30‐01‐2023	362 s
	37.7065 N, 26.9676 E	30‐01‐2023 to 21‐02‐2023	142 s
	37.7068 N, 26.9676 E	21‐02‐2023 to 18‐04‐2023	825 s
	37.7075 N, 26.9673 E	14‐09‐2023 to 27‐09‐2023	105 s

Within the study area, we positioned the camera traps in various strategic locations based on the signs of high activity, such as tracks, scats, prey remains, etc. They were fixed to a tree between 0.20 and 0.30 m from the ground while facing the area of interest. Using this approach, we have positioned the camera traps in altogether 11 different locations. Every 2 weeks, we visited the sites to replace SD cards and batteries and to change the location of the camera traps if the results were not as desired. Data collected by the camera traps consisted of 30 s videos.

Furthermore, coordinates of scats (for their positioning within the landscape and the scats morphology, see Hatlauf, Böcker et al. 2021 supplements) were collected during additional sporadic searches in the study area to estimate the studied group's territory size.

### 2.3 Data Analysis

We analysed the camera trap recordings using an ethogram that we have adapted based on observations on zoo‐housed golden jackals (Table 2) (Wandrey 1975).

**TABLE 2:** Ethogram used in the analysis of the camera trap recordings.

Behaviour	Description
Solitary travel	The observed animal is moving continuously in a specific direction while there are no other individuals visible in the recording
Social travel	Two or more animals are moving as a group continuously in a specific direction within the same recording
Solitary resting	The observed animal is sitting, lying down or standing still in the same location for at least 30 s while there are no other individuals visible in the recording
Social resting	Multiple animals are sitting, lying down or standing still in the same location for at least 30 s (within the same recording)
Solitary foraging/feeding	The observed animal is looking for food or eating while there are no other individuals visible in the recording
Social foraging/feeding	Multiple animals are together looking for food or eating (within the same recording)
Solitary vocalisation	The observed animal is emitting sounds such as barks, howls or yips while there are no other individuals visible in the recording
Social vocalisation	The observed animals are emitting sounds such as barks, howls or yips while there is at least one other individual visible in the recording
Scent marking	The observed animal is applying scent marks by urinating, defecating or rubbing its body against an object
Scanning environment	The observed animal is intensely smelling, listening and or looking around
Affiliation	The observed animals are engaging with each other in a friendly, peaceful and/or playful manner
Aggression	One animal is chasing, biting, growling towards and/or attacking another individual
Submission	One animal is approaching or greeting another individual with a lowered, tucked or low and fast‐wagging tail while showing its bare teeth and flattening its ears

Using the footage collected during the study period, we started identifying individuals based on the colouration patterns of their coats (Macdonald 1979). First, we made a catalogue containing clear images of each individual recorded from different angles and in different light conditions. This catalogue allowed us to identify the individuals within other camera trap recordings when characteristics were visible. We tested for intra‐observer reliability (or observer's bias) by re‐identifying randomly selected camera trap recordings in which a total of 120 individual golden jackals were visible. After re‐identification, we calculated Cohen's Kappa (Cohen 1968; Gammell et al. 2003). Additionally, we tested for temporal independence between camera trap recordings using an autocorrelation function (Chatfield 2004). During the analysis of the camera trap footage, we focussed on social relationships between the group members by looking at social behaviours such as aggression, submission and other behaviours that reveal something about their position within the social hierarchy.

We used records of three of these behaviours to construct a dominance matrix for the group. The behaviours we considered were aggression, submission and scent marking. For aggression, one ‘win’ was added to the dominance matrix in favour of the aggressor towards the recipient of the aggressive behaviour. For submission, one ‘win’ was added in favour of the recipient towards the dominant individual. Finally, for scent marking, one ‘win’ was added in favour of the individual who applied the scent mark towards the rest of the group. Within the dominance matrix, each ‘win’ resulted in an added point of one, while ‘no win’ resulted in no change of points. Based on this dominance matrix, we calculated the normalised David's scores for all adult individuals and used them as a representation of the relative hierarchical rank of the individual group members (David 1987, 1988; Gammell et al. 2003).

During the observation period, we kept a journal describing all interesting events. These events included behavioural changes, appearances of unfamiliar golden jackals, wounded or injured individuals, reproductive behaviour, etc. Coordinates of scent markings were plotted in QGIS (QGIS.org 2023) and used to estimate the boundaries of the group's territory (Figure 3).

Furthermore, we have conducted a social network analysis (SNA) based on the frequencies that individuals were observed in close proximity to each other. SNA is an effective way to describe the social relationships of individuals within a group (Wey et al. 2008). Using the ‘igraph’ package (Csardi and Nepusz 2006) in RStudio (RStudio Team 2020), we have constructed two sociograms. One visualises the social structure of the group before the birth of new pups and the dispersion of several subordinate group members (total of 635 observations), and the other visualises the social structure after these events (total of 406 observations). For each sociogram, we derived two metrics to quantify the structure of the network: eigenvector centrality values of the individual golden jackals and network modularity. The eigenvector centrality values quantify each individual's influence on the whole group based on both their direct and indirect connections with other individuals. Meanwhile, modularity is a measure quantifying the strength of the division of a network into two separate clusters. In the first sociogram, it becomes apparent that the dominant pair has been observed together in considerably more instances than any of the other golden jackals (Wey et al. 2008).

Within the sociogram, nodes represent individual group members, except for the sociogram after the birth of the pups and the dispersion, where all five of the pups are represented by one single node. Node colour indicates sex (white for females, black for males and grey for unknown). The edges linking the nodes present the weighted quality of undirected social relationships between individuals based on the frequencies of association. The thickness of the edges represents the relationship strength calculated using the association index. Eigencentrality values of individual nodes were calculated to quantify the direct and indirect influence of specific individuals on the group. High values indicate a high number and strength of connections (Newman 2004).

In addition, we have determined clusters of golden jackals with stronger relationship weights using the fast greedy algorithm (Clauset, Newman, and Moore 2004). This algorithm uses hierarchical agglomeration to detect structures in a community by greedily optimising the network modularity. The modularity of a network can be calculated by subtracting the fraction of associations within subgroups from the expected fraction, given an equivalent network with a known number of associations from each individual (Newman 2006). Subgroups that have strong ties among their members generally have higher modularity values, whereas low values indicate more homogeneous networks. In practice, values greater than the threshold of 0.3 indicate a strong community structure within a social network (Newman 2004).

## 3 Results

Over 9 months (from 12 December 2023 to 30 October 2023), we have obtained a total of 1564 recordings containing golden jackals. We did not have any recordings for the month of May due to logistical issues. Of the 1.564 recordings, 609 were excluded due to bad video quality, making it impossible to identify individuals. Within the recordings, golden jackals were in frame for 7 h, 29 min and 22 s. The calculated autocorrelations of the footage lie between −0.002 and 0.058, indicating temporal independence of the data (Chatfield 2004).

### 3.1 Individual Characterisation

**FIGURE 4:**
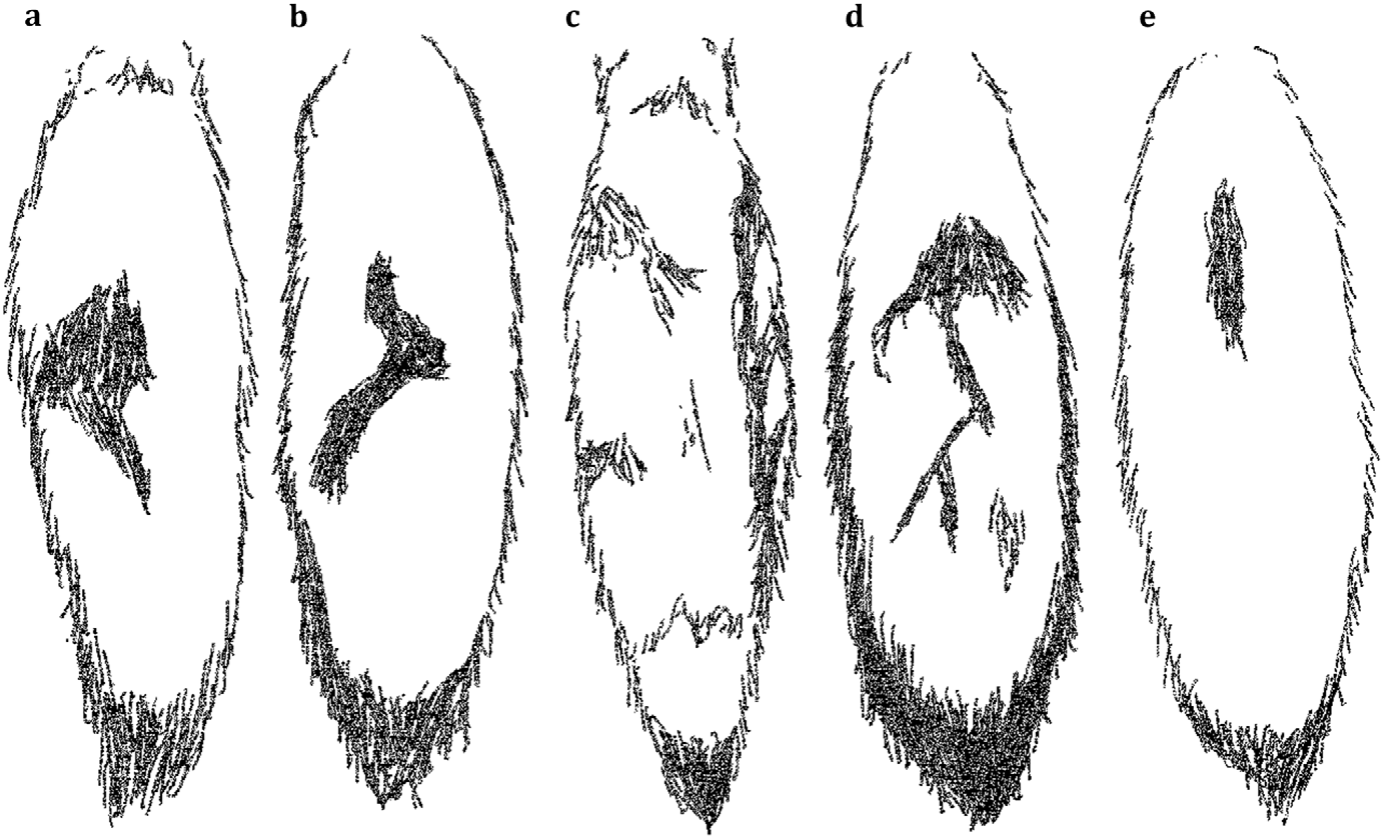
The backs of the tails of the five subordinate members of the studied group, with (a) ‘EC’, (b) ‘SE’, (c) ‘AT’, (d) ‘ME’ and (e) ‘MO’. Individuals can be recognised based on the place and shape of the dark patches on the back of the tail.

**FIGURE 5:**
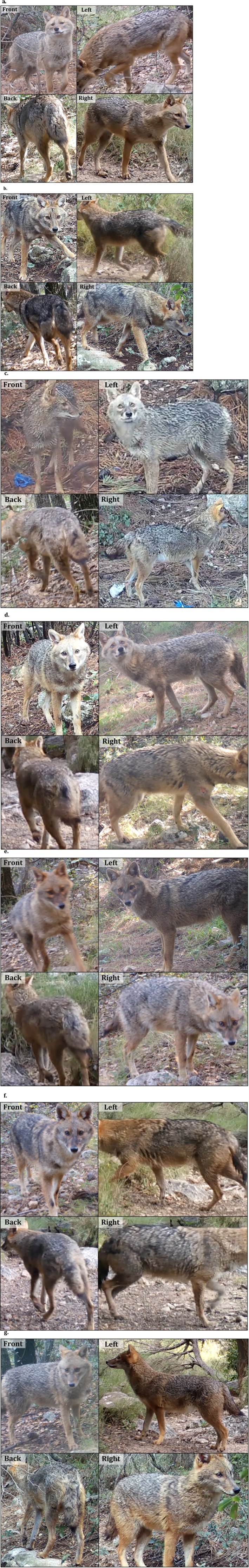
Snapshots taken from camera trap footage showing the individual golden jackals from all angles identified within the studied group, with (a) ‘IR’, (b) ‘TA’, (c) ‘SE’, (d) ‘EC’, (e) ‘MO’, (f) ‘ME’ and (g) ‘AT’.

Individuals were found to indeed vary in coat colouration, making individual identification possible. Main discriminative characteristics were black spots on the front legs, colouration patterns on the chest and dark patches on the back of the tails (Figure 4). Moreover, some individuals had notably lighter or darker coat colours, and distinct marks and patches (cf. Figure 5). The total number of characterised individuals is supported by camera trap videos in which all group members were recorded. The average number of individuals per recording is 1.42. In some cases, we were not able to distinguish individual characteristics (e.g., bad light conditions, fast movement speed, bad camera angle, etc.). Finally, we identified the recorded individuals in 64.4% of the cases. Moreover, we reached a substantial intra‐observer reliability score (*κ* = 0.804). For some of the individuals, we also managed to determine the sexes based on the footage of scent‐marking behaviour and morphological characteristics (Table 3).

**TABLE 3:** Summary of individual group members.

Individual ID	Live stage	Sex	Rank	NormDS
IR	Adult	♀	1	4.655159
TA	Adult	♂	2	4.655159
ME	Adult	♂	3	3.382937
MO	Adult	Na.	4	2.285714
SE	Adult	♀	5	2.142857
EC	Adult	♂	6	2.142857
AT	Adult	Na.	7	1.571429
P1	Juvenile	Na.		
P2	Juvenile	Na.		
P3	Juvenile	Na.		
P4	Juvenile	Na.		
P5	Juvenile	Na.		
P6	Juvenile	Na.		

### 3.2 Social Organisation

**FIGURE 6:**
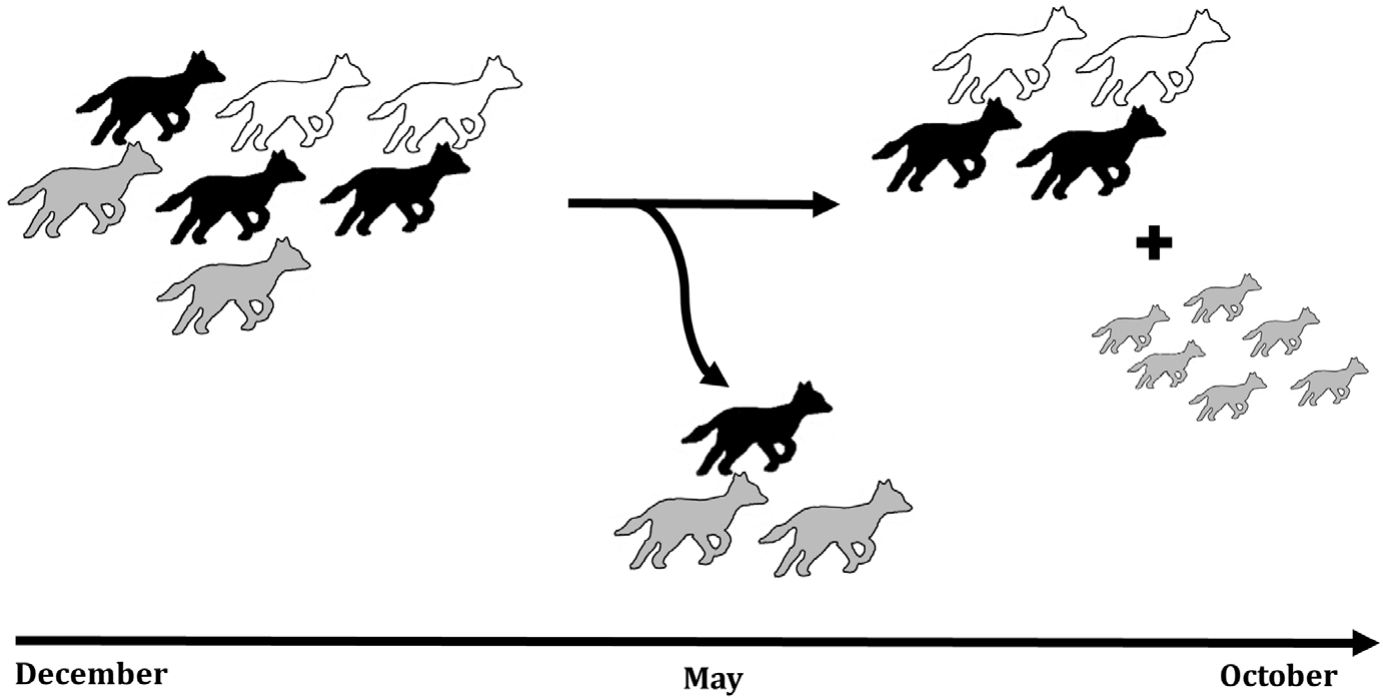
Group composition over time. Colour is indicative for sex (black = male; white = female; grey = unknown) and size for life stage (large = adult; small = juvenile). During the research period, the group composition changed once between April and June, when three group members left and six pups were born.

We found that the studied group had a relatively stable composition. At the start of the study period, it consisted of seven individuals: two females, three males and two of unknown sex. The social group remained stable for 4 months (until May). At this time, a major change occurred involving the dispersion of three individuals and later the birth of a total of six pups (between 13 April and 23 June). After this point, the group remained stable in size and composition for the rest of the study period (Figure 6) (For a video containing all group members after the birth of the pups, see Video 1). From the original five subordinate golden jackals, two individuals, one female and one male, stayed with the group. We have observed them near the pups and engaging with them in social play, suggesting their role as helpers at the nest (Malcolm and Marten 1982).

**VIDEO 1:** Video containing all group members in September 2023. Video content can be viewed at https://onlinelibrary.wiley.com/doi/10.1002/ece3.70620

**FIGURE 7:**
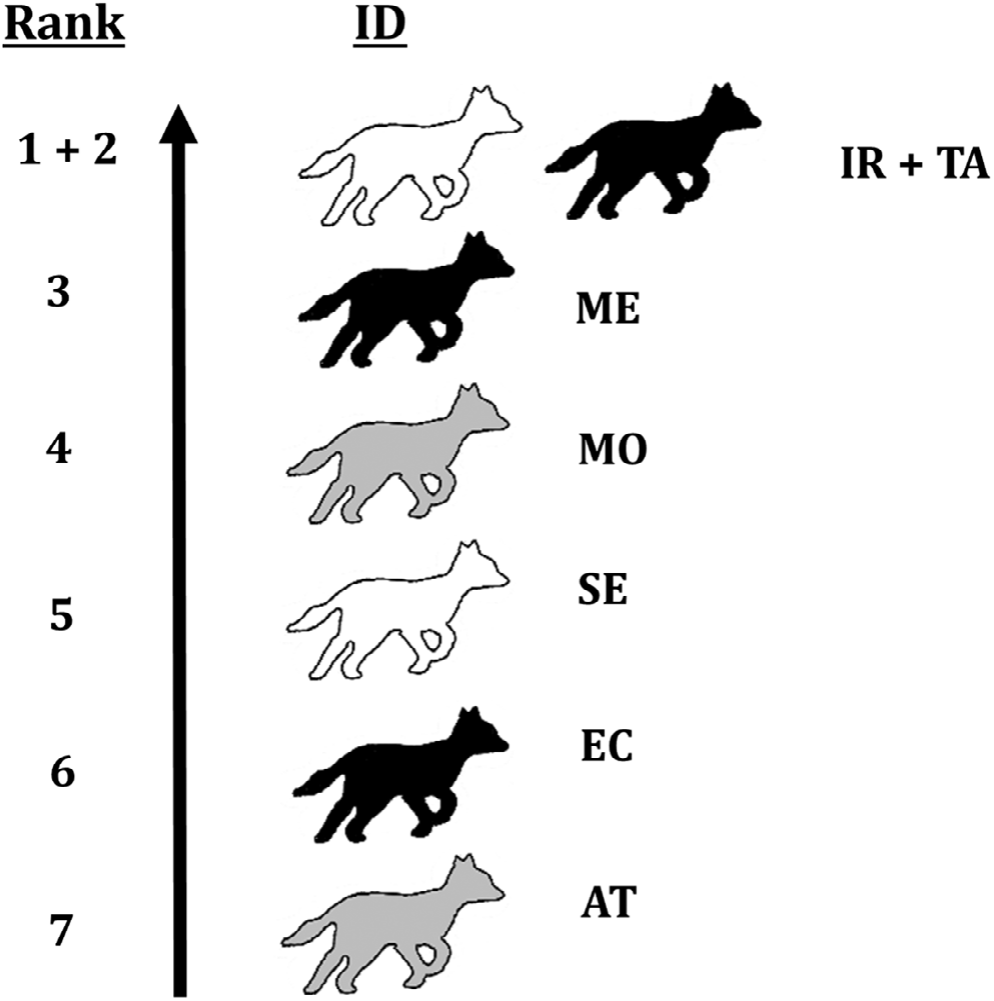
The group's social hierarchy based on the normalised David's score calculated based on aggressive, submissive and scent‐marking behaviour. Colour is indicative for sex (black, male; white, female; grey, unknown).

Within the social group, there were two clear dominant individuals, one female (IR) and one male (TA). Both have the highest normalised David's scores, with the female's score being slightly higher than the male's. Between the rest of the group members, values of normalised David's scores are lower and more homogeneous, indicating equal subordinate positions within the hierarchy (Table 3 and Figure 7) (for an example of behaviour related to dominance, see Video 2).

**VIDEO 2:** Video showing the aggressive behaviour of one individual to the other, and the returned submissive behaviour. Video content can be viewed at https://onlinelibrary.wiley.com/doi/10.1002/ece3.70620

The group had a fixed territory over time estimated to be about 0.07 km^2^ in which they were most active, especially during the hours of daylight (Figure 3). It is within this territory that they engage in nightly group howling ceremonies and in which we have observed them raising their pups.

### 3.3 Mating System

**FIGURE 8:**
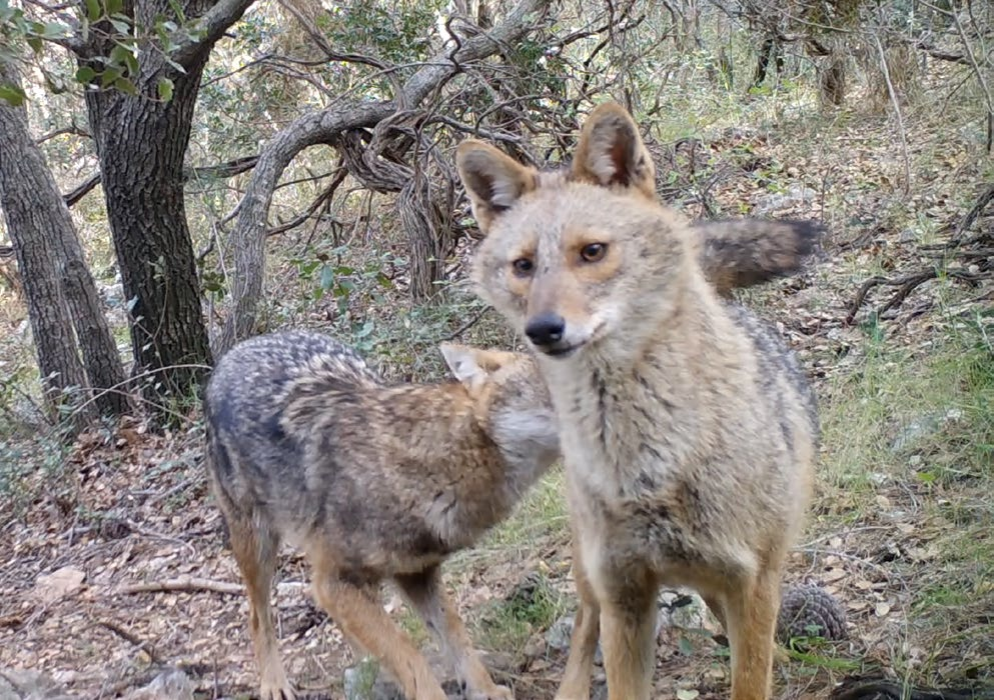
Snapshot taken from camera trap footage showing the dominant male (TA) sniffing and licking the anogenital region of the dominant female (IR), a behaviour associated with pre‐copulation (Wandrey 1975). This footage was recorded on 27 February 2023.

**FIGURE 9:**
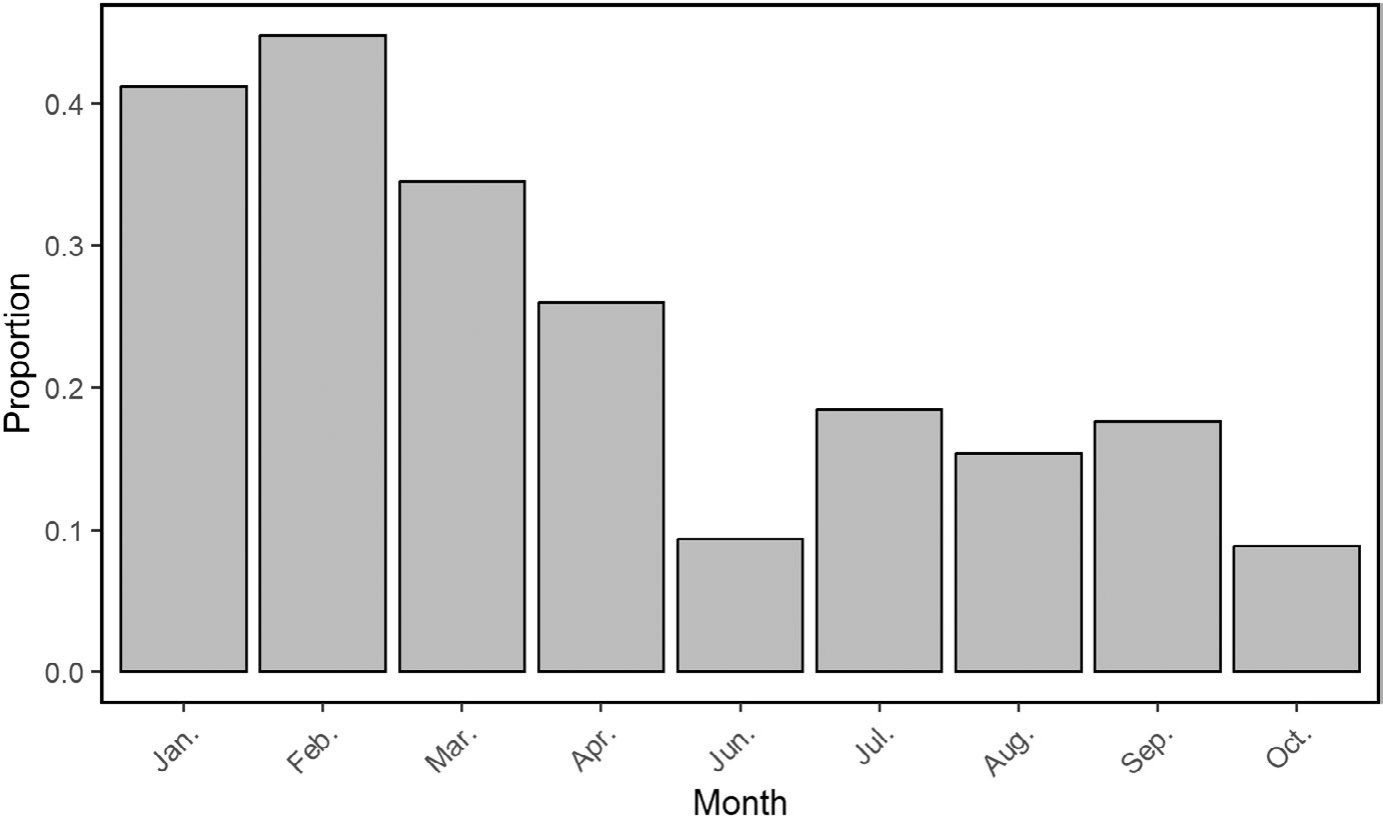
The proportion of recordings of the dominant female and male being in close proximity to each other over the months of the study period (Jan.: 14/34 Feb.: 56/125, Mar. 40/116, Apr.: 20/77, Jun.: 3/32, Jul.: 7/38, Aug.: 2/13, Sep.: 6/34, Oct.: 3/34). Note that we do not have any data for the month of May, as we did not record any data during this month due to logistical issues.

Although the studied group consisted of multiple reproductively mature individuals at all times during the study period, only the dominant female and male have been observed engaging in behaviour related to pre‐copulation (at the start of March). In this instance, the male was sniffing and licking the anogenital area of the female (Figure 8 and Video 3) (cf. Wandrey 1975). We did not observe the mating itself. During the month preceding this event, as well as during the same period, we observed a relatively higher proportion of the dominant pair staying in close proximity to each other (Figure 9). Additionally, we constantly observed the dominant male applying scent marks in the same spot as the dominant female.

**VIDEO 3:** Video showing the anogenital licking between the dominant male and female, an interaction related to pre‐copulatory behaviour (Wandrey 1975). Video content can be viewed at https://onlinelibrary.wiley.com/doi/10.1002/ece3.70620

**FIGURE 10:**
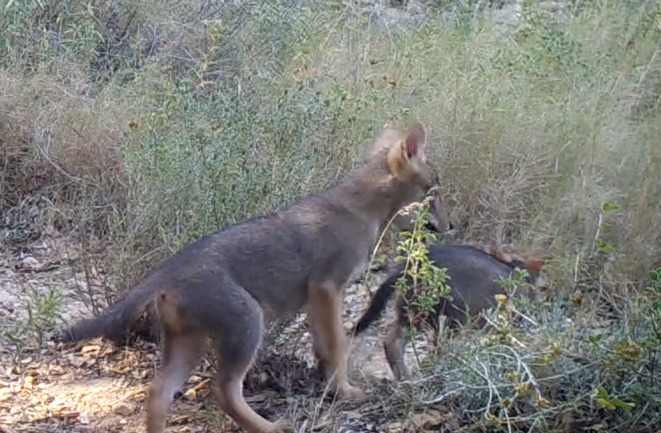
Snapshot taken from camera trap footage showing two pups with a notable size difference between them.

**FIGURE 11:**
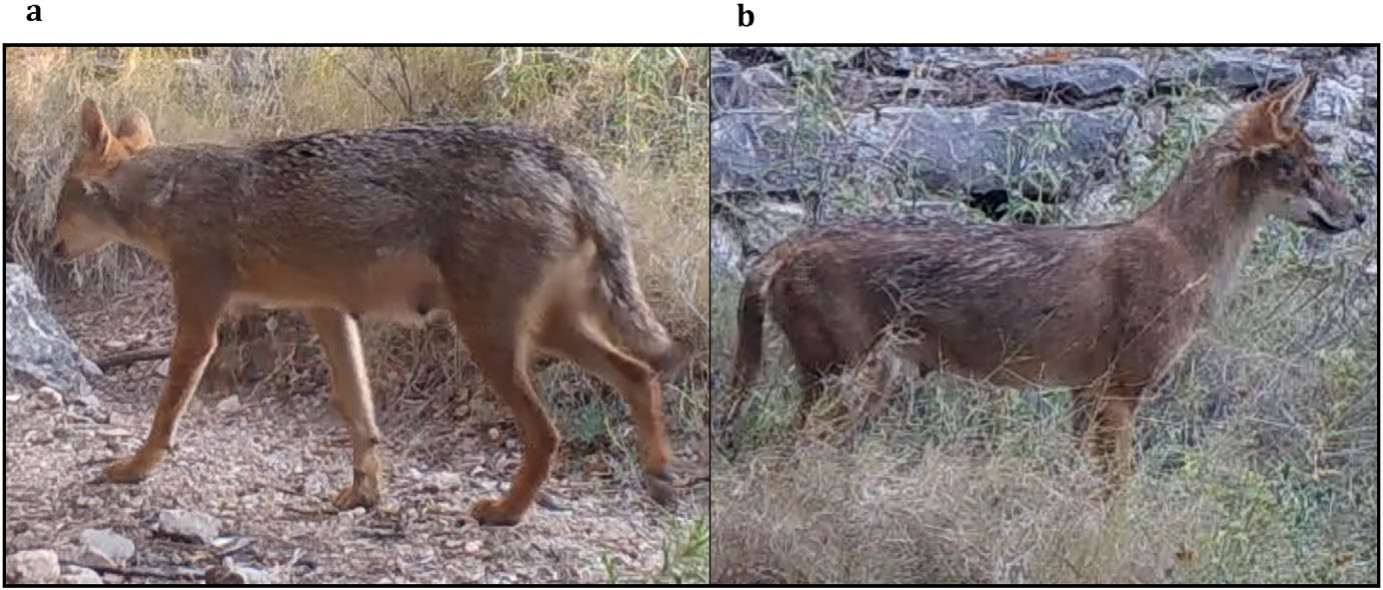
Camera trap images of both females with enlarged nipples, with (a) the dominant female (IR) and (b) the subordinate female (SE).

Moreover, in March, we recorded seven occasions in which unknown golden jackals entered the group's territory. Meanwhile, we did not observe any intruders outside the mating season. We recorded the resident golden jackals meeting the intruders only once. In this interaction, the resident individuals responded by quickly chasing the intruders away. In footage recorded in April, the dominant female appeared to be pregnant. However, it was not until the second half of June that six newborn pups were recorded for the first time. Of these pups, only five were recorded simultaneously at a later time. We observed notable size differences between some of these pups (Figure 10). At this time, the dominant female had enlarged nipples, indicating active suckling. Interestingly, similar to the dominant female, the subordinate female had enlarged nipples (Figure 11).

### 3.4 Social Structure

**FIGURE 12:**
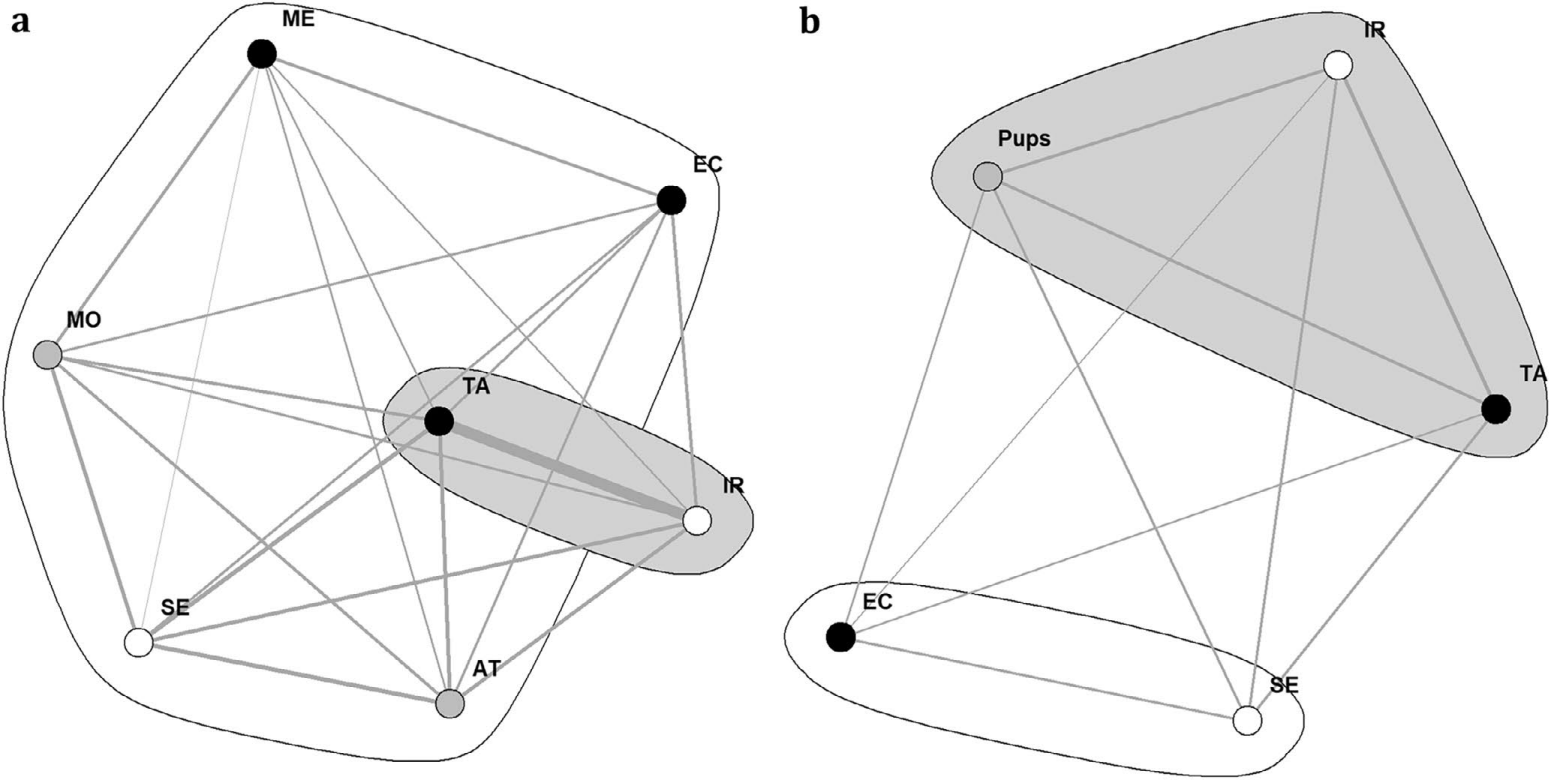
Sociograms based on the number of recorded associations between the jackals from the studied group (a) before (modularity = 0.0735) and (b) after a major shift in the group structure involving the dispersion of three individuals (AT, ME, MO) and the birth of pups (Pups) (modularity = −6.93 × e^−18^). Nodes represent individual jackals (white, dominant female; black, dominant male; grey, subordinate individual, unknown sex). Clusters of individuals with stronger associations are represented by irregular shapes coloured in white and grey (figure was made using the igraph R package).

The results of the social network analyses show that in both networks (before and after dispersion and the birth of the pups), the dominant female (IR) and male (TA) have the highest eigenvector centrality scores, indicating that these were the most centralised individuals within the group (Figure 12). Nevertheless, the pups had a similarly high eigenvector centrality score in the second network. Thus, similar to both dominant individuals, the pups had a central position within the group's social network.

Accordingly, two clusters were recognised within the sociogram, with one containing the dominant female and male and the other containing all of the subordinate group members. The modularity of the social network had a value of 0.05 (Tables 4 and 5), which were below the threshold of 0.3 (30%) (Newman 2006), thus indicating a weak community structure. Therefore, the data only weakly supported the clusters within this sociogram. In the second sociogram, again, two clusters were recognised, with one containing both dominant individuals as well as the pups, and the other cluster containing the two subordinate golden jackals. However, since modularity (−6.93 × *e* ^−18^) was very close to zero (Tables 4 and 5), the clusters within the network were not much different from what would be expected by random chance and were thus only very weakly supported.

**TABLE 4:** Eigenvector centrality scores of the individual jackals and modularity scores of the studied group, displaying the values before a major change in group structure involving the dispersion of three individuals (AT, ME, MO) and the birth of six pups (Pups).

Individual	Eigenvector centrality score
Dominant female (IR)	0.9747901
Dominant male (TA)	1.0000000
Subordinate 1 (ME)	0.3659128
Subordinate 2 (MO)	0.5523451
Subordinate 3 (SE)	0.6491962
Subordinate 4 (EC)	0.4826284
Subordinate 5 (AT)	0.6421497
Modularity	0.05000559

**TABLE 5:** Eigenvector centrality scores of the individual jackals and modularity scores of the studied group, displaying the values after a major change in group structure involving the dispersion of three individuals (AT, ME, MO) and the birth of six pups (Pups).

Individual	Eigenvector centrality score
Dominant female (IR)	1.0000000
Dominant male (TA)	0.9734171
Subordinate 3 (SE)	0.850765
Subordinate 4 (EC)	0.6743985
Pups (Pups)	0.9323426
Modularity	−6.938894E‐18

## 4 Discussion

In this research, close monitoring of a golden jackal group living on Samos using camera trap footage has allowed us to reveal aspects of each of the three interrelated components of its social system: social organisation, mating system and social structure. Successful identification of individual group members based on physical characteristics has proven pivotal to our results. We have found evidence supporting current knowledge on golden jackal social behaviour while gaining some novel insights. The group composition of the studied group coincides with what is to be expected from a golden jackal group, with the group consisting of a dominant pair, a litter of pups, optionally one or more adult (subordinate) helpers and dispersing adults (Macdonald 1979; Moehlman and Hayssen 2018; Wandrey 1975). The fixated behaviour of the dominant male towards the dominant female and the scent‐marking of the same location in quick succession are both indications of hormonal changes occurring in the dominant female that are related to the proestrus phase (Nagashima and Songsasen 2021; Wandrey 1975). The dominant male's behaviour towards the female likely serves as a signal to keep other males from mating with her (Moehlman 1987; Moehlman and Hayssen 2018). The fact that we have observed both this behaviour and anogenital licking between these two individuals only implies monogamy, as is the standard for golden jackals (Macdonald 1979; Moehlman and Hayssen 2018).

Over the study period, the group composition varied, with some yearlings dispersing from their natal group, while two yearlings, a male and a female, stayed at the nest, six pups were born in late spring. Although the exact dates of dispersal and the birth of the pups are unknown, the data suggest that both events roughly concurred. Further, we have determined a clear difference in dominance between two individuals and the rest of the group.

Behavioural changes starting in January suggested that the female was entering proestrus, a key phase before mating (Moehlman 1987; Nagashima and Songsasen 2021). These behavioural changes included the dominant male spending a larger proportion of time than usual in proximity to the dominant female. Moreover, the male immediately marked the same spot whenever the female applied her scent mark. We have observed one case of anogenital licking between the dominant male and female, a behaviour associated with pre‐copulation (Wandrey 1975) (Video 2). The pups were likely born during May, since they were first observed in June (Kebede 2017; Wandrey 1975). At this time, the clearly enlarged nipples of the dominant female indicated active suckling (Jirků et al. 2018). Interestingly, like the dominant female, the subordinate female who stayed as a helper at the nest showed signs of enlarged nipples. However, except for the size difference between pups, there were no indications that she had given birth to pups herself.

SNA has revealed the strengths of the different social relationships within the group, which golden jackals occupy the most influential positions and whether there are clusters or subgroups of individuals within the group. The dominant pair was linked by the strongest tie. Initially, the rest of the network appeared to be relatively homogeneous, with social bonds between subordinates and between dominants and subordinates being roughly equal in strength. However, the birth of the pups and the dispersal of the yearlings have caused a major shift within the network. Afterwards, the pups occupied a central position within the group's social network similar to both dominant individuals. In addition, of both remaining subordinates, the female occupied a more central position than the male. This is a result of her being observed more time babysitting the pups, thus being in a social context. In both analyses, low modularity indicates that clusters within the network are only weakly supported by the data.

Enlargement of the nipples of the female helper without an apparent cause may be the result of a pseudopregnancy (De Vos 1969). Following the ovulation in the absence of mating, female jackals may behave as if they were impregnated. This behaviour is often accompanied by morphological changes including enlargement of the mammary glands, which may secrete a watery murky fluid (De Vos 1969). Hormonal shifts causing such changes could be tied to the strong social hierarchy and reproductive suppression often seen in subordinate females of social canids (Creel and Creel 2002). It is unclear whether this female also attempted to take part in ‘lactating’ the pups, as we have no recordings of any of the females lactating. Another possible explanation may be the observation of a second case of allosuckling in golden jackals in Europe (the first one, cf. Pecorella et al. 2023). This indicates that there would not be one but two litters of pups, with the dominant female being the mother of one and the helper female being the mother of the other litter. Two separate litters would also explain the observed size differences between some of the pups, although this could also be the result of differences in growth rates depending on the amount of energy consumed during the first weeks of their lives (Western 1979). Further research is needed to confirm any of these possible hypotheses.

This is the first time that social network analysis has been conducted on wild golden jackals. The strong tie between the dominant male and female was to be expected, as in golden jackals, the dominant pair generally has a strong pair bond, which lasts for a lifetime. Especially during the mating season, they spend the majority of their time together (Moehlman and Hayssen 2018; Wandrey 1975). Except for the adult pair, the golden jackals in the group do not have a major preference for associating with specific individuals. The clear difference between the dominant pair and other group members reflects the findings of previous studies on hierarchical structures in golden jackal groups (Macdonald 1979; Moehlman 1987). This study adds a new layer of understanding by using SNA, an analysis that has not previously been applied to this species in the wild. This method has allowed for a quantification of the central role of the dominant pair within the group. The finding that the strength of social bonds involving subordinates was roughly equal before the pups were born aligns with the general egalitarian nature of interactions seen in social carnivores (Creel and Creel 2002). The shift within the network after the birth of the pups is mainly the result of all individuals devoting large proportions of their time to providing care to the pups and ‘babysitting’ (Moehlman and Hayssen 2018; Pecorella et al. 2023; Wandrey 1975). The central role of the pups and the increased prominence of the female helper (likely due to her time spent babysitting) illustrate how parental care is a central organising principle in golden jackal societies, especially during pup‐rearing periods (Moehlman 1987).

The lack of clusters within both social networks is likely the result of both networks consisting of only a few individuals who have all been observed interacting with each other. This differs from observations in canid species with larger social groups, where clear clusters of subgroups form within the group's social network (e.g., wolves, Mech 1999). The low modularity observed here implies that only weak subgrouping occurs. Although the frequency of interactions may not be equal between individuals, interactions do occur between all group members (Newman 2006).

Unfortunately, logistic and practical circumstances had not allowed us to continuously monitor the golden jackal group throughout the study period, particularly during May when the pups were likely born. Without continuous monitoring during this time, it is possible that key social dynamics and behavioural changes in the early phase of pup care, such as shifts in the interactions among group members or critical behaviours related to pup‐rearing, may have been missed. The lack of data during May also affects our understanding of seasonal behaviours and their full impact on the social network. While the observed shift in the social network after the birth of the pups suggests significant changes, additional data from the earliest weeks following the pups' birth would have allowed for a more robust assessment of the social interactions and cooperative care roles, contributing to a more comprehensive view of how the social structure of the group fluctuates throughout the year. Although camera traps have provided valuable data, this data collection method limits the detection of more fine‐scale social interactions and dynamics that may occur out of view. More camera traps would have been desirable, as they would have helped to capture more comprehensive data on individual interactions and social changes within the group. Additionally, it would have been valuable to use genetic tests to confirm whether the pups are indeed of separate litters. Such tests could provide clearer answers about allosuckling or multi‐litter group structures, as has been observed in golden jackals before (Pecorella et al. 2023).

## 5 Conclusion

In this study, we aimed to uncover various aspects of the social system of one golden jackal group using a relatively non‐invasive method. With a limited number of camera traps, we successfully identified individual group members for the first time in Europe, revealed their social relationships and monitored changes within the group throughout the year. In doing so, we proved that golden jackals can indeed be distinguished based on unique physical characteristics. As predicted, our findings support and refine our current general knowledge of golden jackal social behaviour. Moreover, our predictions regarding variation in group composition and social behaviour over time have also been proven correct.

Despite some limitations, we accomplished our goal of gaining new insights into the social behaviour of the unique population of golden jackals living on Samos Island. A key limitation was the limited number of camera traps, which restricted our ability to capture fine‐scale data on individual interactions. Additionally, the absence of data in May, a critical period for pup birth and early care, has constrained our understanding of the social dynamics during this pivotal time. Nevertheless, our findings contribute to our broader knowledge of golden jackal social behaviour, as we have confirmed some general topics while revealing new insights. We hope this knowledge will contribute to a better understanding of the species and their social interactions, which are important in developing specific and effective conservation methods.

In the future, it would be desirable to repeat a similar study over a continuous period using more camera traps. This would allow for better and easier identification of individual golden jackals, and closer monitoring would give a more complete representation of the social interactions between individuals, especially during the mating season and while raising pups. In addition, using molecular techniques would allow us to confirm the relatedness of the group members.

## Author Contributions

**Jonas Custers:** conceptualization (lead), data curation (lead), formal analysis (lead), investigation (lead), methodology (lead), project administration (equal), resources (lead), validation (equal), visualization (equal), writing – original draft (lead), writing – review and editing (lead). **Jennifer Hatlauf:** funding acquisition (equal), methodology (equal), supervision (lead), writing – review and editing (equal). **Sem van der Niet:** data curation (equal), investigation (equal), project administration (equal), validation (equal), writing – review and editing (equal). **Beatriz Tintoré:** conceptualization (equal), methodology (equal), project administration (equal), supervision (equal), writing – review and editing (equal). **Anastasia Miliou:** conceptualization (equal), project administration (equal), resources (equal), supervision (equal), validation (equal).

## Conflicts of Interest

The authors declare no conflicts of interest.

## Data Availability

The data that support the findings of this study are openly available in OSF at https://osf.io/ntfjc/?view_only=853c6266aa624c1cb883700956bf2dd4.

## References

[ece370620-bib-0001] Berger‐Tal, O. , and D. Saltz . 2016. Conservation Behavior, Applying Behavioral Ecology to Wildlife Conservation and Management. Cambridge, UK: Cambridge University Press.

[ece370620-bib-0002] Blakeslee, A. M. H. , L. E. Haram , I. Altman , K. Kennedy , G. M. Ruiz , and A. W. Miller . 2019. “Founder Effects and Species Introductions: A Host Versus Parasite Perspective.” Evolutionary Applications 13, no. 3: 559–574. 10.1111/eva.12868. 32431736 PMC7045715

[ece370620-bib-0003] Bouchet, S. T. 2017. “Le chacal doré observé pour la première fois en France.” Le dauphiné. https://www.ledauphine.com/insolite/2017/12/14/le‐chacal‐dore‐observe‐pour‐la‐premiere‐fois‐en‐france.

[ece370620-bib-0004] Bro‐Jørgensen, J. , D. W. Franks , and K. Meise . 2019. “Linking Behaviour to Dynamics of Populations and Communities: Application of Novel Approaches in Behavioural Ecology to Conservation.” Philosophical Transactions of the Royal Society of London. Series B, Biological Sciences 374: 20190008. 10.1098/rstb.2019.0008. 31352890 PMC6710565

[ece370620-bib-0005] Bulmer, L. 2015. “The Impact of Anthropogenic Disturbance on the Behaviour and Ecology of the Golden Jackal ( *Canis aureus* ).” Doctoral dissertation, University of York.

[ece370620-bib-0006] Buruaga, M. , J. Carreras , M. Madeira , M. Olalde , C. Mártioda , and M. Campos . 2023. “Primera cita de chacal dorado ( *Canis aureus* ) en la península Ibérica.” Galemys, Spanish Journal of Mammalogy 35: 1–3. 10.7325/Galemys.2023.N5.

[ece370620-bib-0007] Chatfield, C. 2004. The Analysis of Time Series: An Introduction. 6th ed. Boca Raton: Chapman and Hall/CRC.

[ece370620-bib-0008] Ćirović, D. , A. Penezić , and M. Krofel . 2016. “Jackals as Cleaners: Ecosystem Services Provided by a Mesocarnivore in Human‐Dominated Landscapes.” Biological Conservation 199: 51–55. 10.1016/j.biocon.2016.04.027.

[ece370620-bib-0009] Clauset, A. , M. E. J. Newman , and C. Moore . 2004. “Finding Community Structure in Very Large Networks.” Physical Review E ‐ Statistical Physics, Plasmas, Fluids, and Related Interdisciplinary Topics 70, no. 6: 6. 10.1103/PHYSREVE.70.066111/FIGURES/3/MEDIUM. 15697438

[ece370620-bib-0010] Clutton‐Brock, T. H. 1989. “Review Lecture: Mammalian Mating Systems.” Proceedings of the Royal Society of London B: Biological Sciences 126, no. 1285: 339–372. 10.1098/RSPB.1989.0027. 2567517

[ece370620-bib-0011] Cohen, J. 1968. “Weighted Kappa: Nominal Scale Agreement With Provision for Scaled Disagreement or Partial Credit.” Psychological Bulletin 70, no. 4: 213–220. 10.1037/h0026256. 19673146

[ece370620-bib-0012] Creel, S. , and N. M. Creel . 2002. The African Wild Dog: Behavior, Ecology, and Conservation, Vol. 65. Princeton, NJ: Princeton University Press.

[ece370620-bib-0013] Csányi, E. , J. Lanszki , M. Heltai , M. Pölös , G. Schally , and G. Sándor . 2023. “The First Evidence of the Monogamous Golden Jackal's Adaptive Response to Partner Loss.” Applied Animal Behaviour Science 269: 106095. 10.1016/j.applanim.2023.106095.

[ece370620-bib-0014] Csardi, G. , and T. Nepusz . 2006. “The Igraph Software Package for Complex Network Research.” InterJournal, Complex Systems (1695). http://igraph.org.

[ece370620-bib-0015] Custers, J. 2022. “Social Culture in Bonobos ( *Pan paniscus* ) and Chimpanzees ( *Pan troglodytes* )?” Master thesis, Utrecht University.

[ece370620-bib-0016] David, H. A. 1987. “Ranking From Unbalanced Paired‐Comparison Data.” Biometrika 74, no. 2: 432. 10.2307/2336160.

[ece370620-bib-0017] David, H. A. 1988. The Method of Paired Comparisons. London: Charles Griffin.

[ece370620-bib-0018] De Vos, V. 1969. “Psuedo‐Pregnancy in the Black‐Backed Jackal ( *Canis mesomelas* Schreber).” Journal of the South African Veterinary Association 40, no. 4: 381–383.

[ece370620-bib-0019] Gammell, M. P. , H. de Vries , D. J. Jennings , C. M. Carlin , and T. J. Hayden . 2003. “David's Score: A More Appropriate Dominance Ranking Method Than Clutton‐Brock et al.'s Index.” Animal Behaviour 66, no. 3: 601–605. 10.1006/ANBE.2003.2226.

[ece370620-bib-0021] Giannatos, G. 2004. Conservation Action Plan for the Golden Jackal ( *Canis aureus* L) in Greece, 47. Greece: WWF.

[ece370620-bib-0022] Giannatos, G. , Y. Marinos , P. Maragou , and G. Catsadorakis . 2005. “The Status of the Golden Jackal ( *Canis aureus* ) in Greece.” Belgian Journal of Zoology 135, no. 2: 145–149.

[ece370620-bib-0023] Hatlauf, J. , K. Bayer , A. Trouwborst , and K. Hackländer . 2021. “New Rules or Old Concepts? The Golden Jackal ( *Canis aureus* ) and Its Legal Status in Central Europe.” European Journal of Wildlife Research 67, no. 25: 1–15. 10.1007/s10344-020-01454-2.

[ece370620-bib-0024] Hatlauf, J. , F. Böcker , L. Wirk , et al. 2021. “Jackal in Hide: Detection Dogs Show First Success in the Quest for Golden Jackal ( *Canis aureus* ) Scats.” Mammal Research 66: 227–236. 10.1007/s13364-020-00537-4.

[ece370620-bib-0025] Hinde, R. A. 1976. “Interactions, Relationships and Social Structure.” Man 11, no. 1: 1–17. 10.2307/2800384.

[ece370620-bib-0026] Jirků, M. , D. Dostál , J. Robovský , and M. Šálek . 2018. “Reproduction of the Golden Jackal ( *Canis aureus* ) Outside Current Resident Breeding Populations in Europe: Evidence From The Czech Republic.” Mammalia 82: 592–595. 10.1515/mammalia-2017-0141.

[ece370620-bib-0027] Kappeler, P. M. , L. Barrett , D. T. Blumstein , and T. H. Clutton‐Brock . 2013. “Constraints and Flexibility in Mammalian Social Behaviour: Introduction and Synthesis.” Philosophical Transactions of the Royal Society, B: Biological Sciences 368, no. 1618: 20120337. 10.1098/rstb.2012.0337. PMC3638441 23569286

[ece370620-bib-0028] Karamanlidis, A. A. , M. de Gabriel Hernando , M. Avgerinou , et al. 2023. “Rapid Expansion of the Golden Jackal in Greece: Research, Management and Conservation Priorities.” Endangered Species Research 51: 1–13. 10.3354/esr01238.

[ece370620-bib-0029] Kebede, Y. 2017. “A Review on: Distribution, Ecology and Status of Golden Jackal ( *Canis aureus* ) in Africa.” Journal of Natural Science Research 7, no. 1: 32–43.

[ece370620-bib-0030] Kojola, I. , H. Henttonen , S. Heikkinen , and N. Ranc . 2024. “Golden Jackal Expansion in Northernmost Europe: Records in Finland.” Mammalian Biology 104: 101–105. 10.1007/s42991-023-00382-3.

[ece370620-bib-0031] Krofel, M. , G. Giannatos , D. Ćirovič , S. Stoyanov , and T. M. Newsome . 2017. “Golden Jackal Expansion in Europe: A Case of Mesopredator Release Triggered by Continent‐Wide Wolf Persecution?” Hystrix, the Italian Journal of Mammalogy 28, no. 1: 9–15. 10.4404/hystrix-28.1-11819.

[ece370620-bib-0032] Lanszki, J. , G. Schally , M. Heltai , and N. Ranc . 2018. “Golden Jackal Expansion in Europe: First Telemetry Evidence of a Natal Dispersal.” Mammalian Biology 88: 81–84. 10.1016/j.mambio.2017.11.011.

[ece370620-bib-0033] Macdonald, D. W. 1979. “The Flexible Social System of Golden Jackal, *Canis aureus* .” Behavioural Ecology and Sociobiology 5, no. 1: 17–38. 10.1007/bf00302692.

[ece370620-bib-0034] Malcolm, J. R. , and K. Marten . 1982. “Natural Selection and the Communal Rearing of Pups in African Wild Dogs ( *Lycaon pictus* ).” Behavioral Ecology and Sociobiology 10, no. 1: 1–13. 10.1007/bf00296390.

[ece370620-bib-0035] Matthews, T. J. , and K. Triantis . 2021. “Island Biogeography.” Current Biology 31, no. 19: R1201–R1207. 10.1016/j.cub.2021.07.033. 34637732

[ece370620-bib-0036] Mech, L. D. 1999. “Alpha Status, Dominance, and Division of Labor in Wolf Packs.” Canadian Journal of Zoology 77, no. 8: 1196–1203. 10.1139/z99-099.

[ece370620-bib-0037] Moehlman, P. D. 1987. “Social Organization in Jackals: The Complex Social System of Jackals Allows the Successful Rearing of Very Dependent Young.” American Scientist 75, no. 4: 366–375.

[ece370620-bib-0038] Moehlman, P. D. , and V. Hayssen . 2018. “ *Canis aureus* (Carnivore: Canidae).” Mammalian Species 50, no. 957: 14–25. 10.1093/MSPECIES/SEY002.

[ece370620-bib-0039] Nagashima, J. B. , and N. Songsasen . 2021. “Canid Reproductive Biology: Norm and Unique Aspects in Strategies and Mechanisms.” Animals 11: 653. 10.3390/ani11030653. 33804569 PMC8001368

[ece370620-bib-0040] Newman, M. E. J. 2004. “Detecting Community Structure in Networks.” European Physical Journal B 38, no. 2: 321–330. 10.1140/EPJB/E2004-00124-Y.

[ece370620-bib-0041] Newman, M. E. J. 2006. “Modularity and Community Structure in Networks.” Proceedings of the National Academy of Sciences of the United States of America 103, no. 23: 8577–8582. 10.1073/pnas.0601602103. 16723398 PMC1482622

[ece370620-bib-0042] Pecorella, S. , M. De Luca , F. Fonda , et al. 2023. “First Record of Allonursing in Golden Jackal ( *Canis aureus* , L. 1758): A Case of Double Breeding and Communal Denning Within the Same Social Unit.” European Journal of Wildlife Research 69: 43. 10.1007/s10344-023-01671-5.

[ece370620-bib-0043] Pietroluongo, G. , J. Leggett , F. J. Falquina Fernández , et al. 2018. “Monitoring of a *Canis aureus* Population Living in the Airport Area of Samos Island, Greece.” Second International Jackal Symposium, Marathon Bay, Attica Greece (October 2018).

[ece370620-bib-0044] Pietroluongo, G. , I. Linardaki , J. Leggett , et al. 2018. “Preliminary Assessment of Feeding Ecology of a Golden jackal ( *Canis aureus* ) Population in South Eastern Samos Island, Greece, Through Post Mortem Examination and Scat Analysis.” 2nd International Jackal SymposiumAt: Samos, Greece. 10.13140/RG.2.2.35441.33120.

[ece370620-bib-0045] QGIS.org . 2023. “QGIS Geographic Information System.” Open Source Geospatial Foundation Project. http://qgis.org.

[ece370620-bib-0046] Royo‐Vicente, A. , and F. J. García . 2024. “Segunda cita de chacal dorado *Canis aureus* en la península ibérica (Zaragoza, Aragón) mediante fototrampeo. Galemys, Spanish.” Journal of Mammalogy 36: 1–2.

[ece370620-bib-0047] RStudio Team . 2020. RStudio: Integrated Development for R. PBC, Boston, MA: RStudio. http://www.rstudio.com/.

[ece370620-bib-0048] Rutkowski, R. , M. Krofel , G. Giannatos , et al. 2015. “A European Concern? Genetic Structure and Expansion of Golden Jackals ( *Canis aureus* ) in Europe and the Caucasus.” PLoS One 10, no. 11: e0141236. 10.1371/journal.pone.0141236. 26540195 PMC4634961

[ece370620-bib-0049] Rykov, A. M. , A. S. Kuznetsova , and K. F. Tirronen . 2022. “The First Record of the Golden Jackal ( *Canis aureus* Linnaeus, 1758) in the Russian Subarctic.” Polar Biology 45: 965–970. 10.1007/s00300-022-03037-0.

[ece370620-bib-0050] Sørensen, O. J. , and L. K. Lindsø . 2021. “The Golden Jackal *Canis aureus* Detected in Norway – Management Challenges With Naturally Dispersed Species New to the Country (Original Title: Gullsjakal påvist i Norge – Forvaltningsutfordringer Ved Nye Arter i Landet).” Fauna 74: 74–87.

[ece370620-bib-0051] Stefanović, M. , W. Bogdanowicz , R. Adavoudi , et al. 2024. “Range‐Wide Phylogeography of the Golden Jackals ( *Canis aureus* ) Reveals Multiple Sources of Recent Spatial Expansion and Admixture With Dogs at the Expansion Front.” Biological Conservation 290: 110448. 10.1016/j.biocon.2024.110448.

[ece370620-bib-0052] Stiros, S. C. , J. Laborel , F. Laborel‐Deguen , S. Papageorgiou , J. Evin , and P. A. Pirazzoli . 2000. “Seismic Coastal Uplift in a Region of Subsidence: Holocene Raised Shorelines of Samos Island, Aegean Sea, Greece.” Marine Geology 170, no. 1–2: 41–58.

[ece370620-bib-0053] Trouwborst, A. , M. Krofel , and J. D. C. Linnell . 2015. “Legal Implications of Range Expansions in a Terrestrial Carnivore: The Case of the Golden Jackal ( *Canis aureus* ) in Europe.” Biodiversity and Conservation 24: 2593–2610. 10.1007/s10531-015-0948-y.

[ece370620-bib-0054] Wandrey, R. 1975. “Contribution to the Study of the Social Behaviour of Captive Golden Jackals ( *Canis aureus* L.).” Zeitschrift für Tierpsychologie 39, no. 1–5: 365–402. 10.1111/J.1439-0310.1975.TB00915.X.

[ece370620-bib-0055] Western, D. 1979. “Size, Life History and Ecology in Mammals.” African Journal of Ecology 17, no. 4: 185–204. 10.1111/j.1365-2028.1979.tb00256.x.

[ece370620-bib-0056] Wey, T. , D. T. Blumstein , W. Shen , and F. Jordán . 2008. “Social Network Analysis of Animal Behaviour: A Promising Tool for the Study of Sociality.” Animal Behaviour 75, no. 2: 333–344. 10.1016/J.ANBEHAV.2007.06.020.

